# Allele-specific polymerase chain reaction for the discrimination of elite Korean cattle associated with high beef quality and quantity

**DOI:** 10.5194/aab-65-47-2022

**Published:** 2022-02-07

**Authors:** Wonhee Lee, Insik Nam, Daehyun Kim, Kukdong Kim, Yoonseok Lee

**Affiliations:** 1 Department of Biotechnology, Hankyong National University, Gyeonggi-Do, Anseong, 17579, Republic of Korea; 2 Center for Genetic Information, Hankyong National University, Gyeonggi-Do, Anseong, 17579, Republic of Korea; 3 Department of Animal Life Convergence Science, Hankyong National University, Gyeonggi-Do, Anseong, 17579, Republic of Korea; 4 Livestock Research Institute, Gyeongsangbuk-Do, Yeongju, 36052, Republic of Korea; 5 Ministry of SMEs and Startups, Chungcheongnam-Do, Chunan, 31169, Republic of Korea

## Abstract

Techniques such as direct sequencing and PCR-RFLP (restriction fragment length polymorphism) are widely used to analyze the genotypes of livestock. However, these conventional methods have the disadvantage of taking a lot of time and incurring considerable cost. The allele-specific PCR method performs PCR using two primers, and a single nucleotide polymorphism (SNP) genotype can be identified through electrophoresis, saving time and cost. Highly accurate results can be obtained by designing specific primers according to the allele of the SNP under study, utilizing primer binding to a complementary matching sequence. In this study, we established a genotyping system with the AS-PCR technique, using SNPs related to the improvement of the meat quality and meat mass of Korean cattle. Using the PRIMER1 program, we designed specific primers for SNPs located at the 3 ${}^{\prime }$ end, with one SNP marker in the *HSPB1* gene related to meat quantity and two SNP markers in the *ADH1C* and *FASN* genes related to meat quality in cattle. AS-PCR was performed on 10 Korean cattle using the primers designed with this system, and the genotypes could be identified by the size of the PCR product amplified as a result of electrophoresis. In the case of the *HSPB1* g.2352T > C SNP, the T allele was amplified to 148 bp, and the C allele was amplified to 222 bp. The *ADH1C* c.-64T > C SNP was amplified to 492 bp at the T allele and 330 bp at the C allele. The *FASN* g.17924G > A SNP A allele was amplified to 377 bp and the G allele to 507 bp. The results for each SNP genotype were verified using direct sequencing, which showed that the genotypes identified by direct sequencing and the genotypes identified by the AS-PCR method matched exactly. The AS-PCR method therefore appears to be valuable for use in a genotyping system.

**Figure 1:**
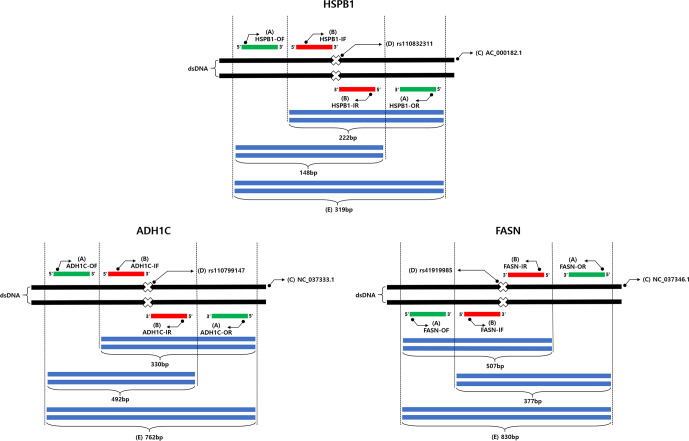
Schematic diagram of AS-PCR amplification and size of fragments. (A) Outer primers are indicated by green rectangles. (B) Inner primers are indicated by red rectangles. (C) DNA sequences were retrieved from NCBI using their accession numbers. (D) The rs numbers of SNPs are indicated by cross marks, and the amplified size of each SNP genotype is indicated by blue rectangles. (E) Amplicon of positive control.

**Figure 2:**
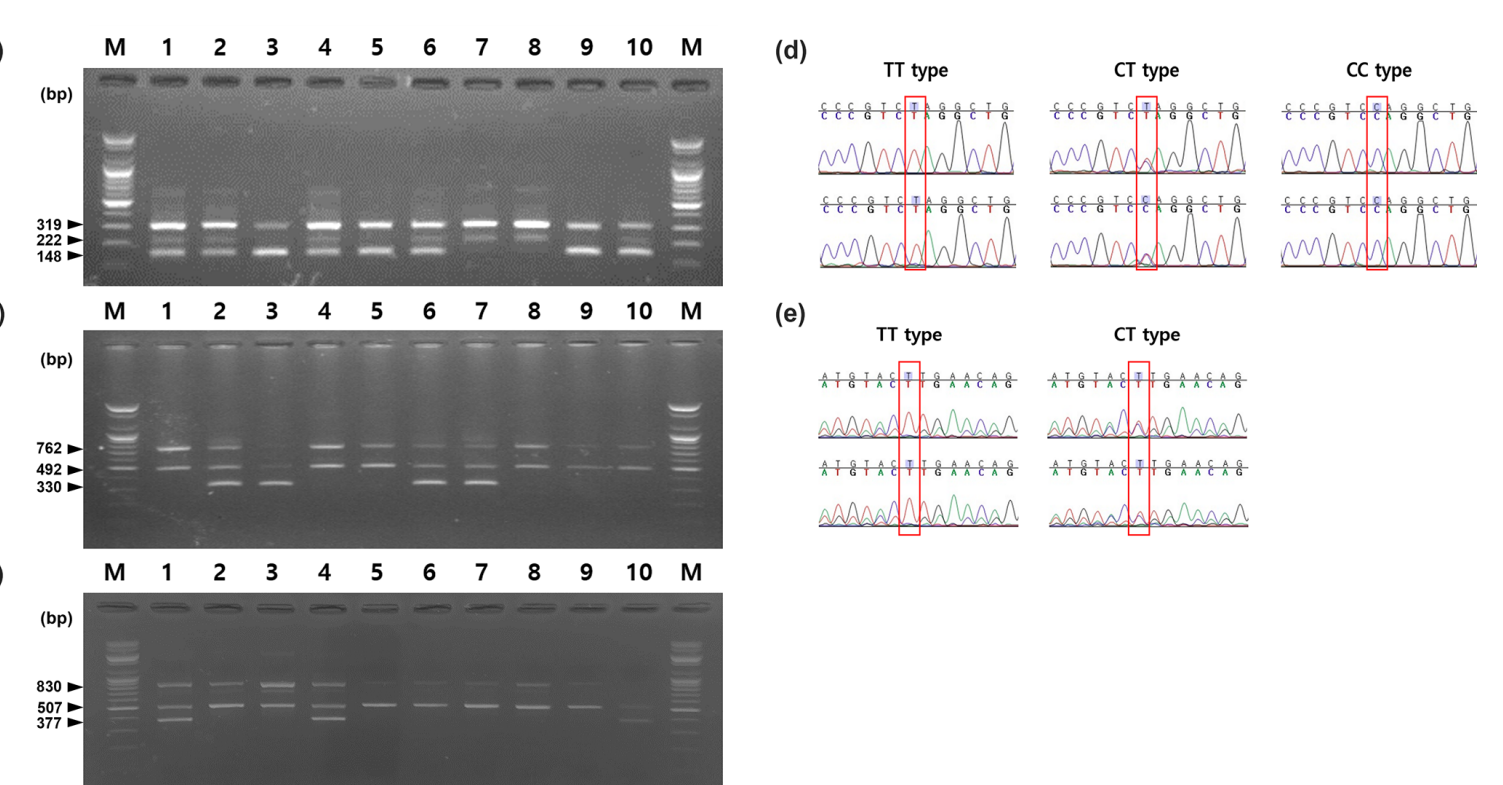
Results of electrophoresis for each genotype amplified using three SNPs. M : molecular weight marker. **(a)** *HSPB1* g.2352T > C SNP, CC type was amplified to 222 and 319 bp, CT type was amplified to 148, 222, and 319 bp, TT type was amplified to 148 and 319 bp; **(b)** *ADH1C* c.-64T > C SNP, TT type was amplified 492, 762 bp, CT type was amplified 330, 492, 762 bp; **(c)** *FASN* g.17924G > A SNP, GG type was amplified 507, 830 bp, AG type was amplified 377, 507, 830 bp; **(d)** result of direct sequencing for the *HSPB1* g.2352T > C SNP; **(e)** result of direct sequencing for the *ADH1C* c.-64T > C SNP.

## 1 Introduction

Recently, research into the development of DNA markers related to economic traits such as meat quantity and quality in cattle has been reported. Among these DNA markers, single nucleotide polymorphisms (SNPs) are single base differences between the DNA of different individuals. SNPs are more suited for use as genotyping markers than conventional markers such as restriction fragment length polymorphisms (RFLPs), amplified fragment length polymorphisms (AFLPs), and simple sequence repeats (SSRs), because SNPs are the most abundant and stable form of genetic variation in the cattle genome. With new developments in biotechnology, SNPs are becoming favored genetic markers for use in marker-assisted selection breeding programs. In a field farm, traditional SNP genotyping methods such as DNA sequencing and PCR-RFLP, have been used to select elite cattle with excellent meat quality and quantity. Usually, these methods are expensive, time-consuming, and labor-intensive and require specialized equipment. There is therefore a need for simple and accurate genotyping assays that can be implemented in laboratories lacking access to sophisticated equipment. Recently, allele-specific PCR (AS-PCR) has been widely used for low-throughput applications in genetic breeding in cattle (Zhao et al., 2019; Darawi et al., 2013). AS-PCR is based on the extension of primers, which only occurs when the 3 ${}^{\prime }$ end is a perfect complement to the template. In principle, SNPs can be detected using allele-specific PCR primers based on the 3 ${}^{\prime }$ -terminal nucleotide of a primer that corresponds to a specific SNP site. However, reliable discrimination between alleles cannot be achieved using this method. To overcome this problem, allele-specific primers with an additional base pair change within the three bases closest to the SNP site between have been used (Cha et al., 1992; Kwok et al., 1994). Considerable research has been conducted using SNPs identified as being related to meat quality and quantity. Zhang et al. (2014) published a study that demonstrated that the expression of the heat-shock protein beta-1 (*HSPB1*) gene induces muscle formation during the growth of beef cattle (Zhang et al., 2014), and a study comparing variations in the weight of Korean cattle according to the SNP genotype of the *HSPB1* gene was published. Kim et al. (2009) reported that the *FASN* (g.17924 G > A) gene is closely associated with fatty acid metabolism, controlling the quality of Korean beef. SNPs can be used as DNA markers for producing high-quality Korean beef as discriminating meat-related genetic factors (Kim et al., 2009). Using the c.-64T > C SNP of the *ADH1C* gene, Ward et al. (2012) reported that marbling in cattle with the TT genotype of the *ADH1C* gene was higher than that in TC and CC genotypes during 5 months of restriction of vitamin A in Angus crossbred steers (Ward et al., 2012). Peng et al. (2017) analyzed the association of the c.-64T > C SNP of the *ADH1C* gene with intramuscular fat deposition in Korean cattle during experiments with vitamin-A restriction in Korean cattle steers (Peng et al., 2017). The aim of this study was to establish a genotyping system based on AS-PCR analysis using three SNPs in the *HSPB1*, *ADH1C*, and *FASN* genes, to make genotyping more convenient and to reduce the amount of labor and time required.

## 2 Materials and methods

### 2.1 DNA preparation

Genomic DNA was extracted from 10 sirloin tissues using G-DEX™ Genomic DNA extraction kits (Intronbio, South Korea). First, 100 mg of Korean beef tissue was added to a 2 mL tube, with 600 µL of lysis buffer and 0.5 µL each of proteinase K and RNase. The lysis process was carried out at 37 ${}^{\circ }$ C overnight. The following day, 300 µL of PPT buffer was added and vortexed, the solution was centrifuged at 12 000 rpm for 10 min, and the supernatant was transferred to a 1.5 mL tube. Then 300 µL of isopropanol was added to the supernatant, which was mixed and stored at -20 ${}^{\circ }$ C for 1 h. The mixture was then centrifuged at 12 000 rpm for 5 min to obtain the DNA pellet, which was washed with 99 % ethanol. The genomic DNA was suspended in 100 µL of TE buffer after the ethanol dried and was stored at -20 ${}^{\circ }$ C for use in this study. The sequences of the extracted DNA were confirmed using electrophoresis.

### 2.2 Primer design and amplification of AS-PCR

In order to conduct AS-PCR for each SNP in the *HSPB1* (g.2352T > C), *ADH1C* (c.-64T > C), *FASN* (g.17924G > A) genes, primers were designed using PRIMER (http://primer1.soton.ac.uk, last access: 15 November 2019). FASTA sequences extracted from Ensemble (http://www.ensembl.org, last access: 15 November 2019) were used. The position of the SNP was marked in the PRIMER1 program so that SNP was located at the 3 ${}^{\prime }$ end of the specific primer. To increase the specificity of the primer, the third nucleotide from the 3 ${}^{\prime }$ end of the specific primer was mismatched. The primers used in this study are shown in Table 1. When AS-PCR was performed using the primer for each gene, the PCR products were amplified to 148 bp for the T allele, 222 bp for the C allele and 319 bp for the control in the *HSPB1* gene. For the *ADH1C* gene, the T allele was amplified to 492 bp, the C allele to 330 bp, and the control to 762 bp. For the *FASN* gene, the A allele was amplified to 377 bp, the G allele to 507 bp, and the control to 830 bp. For the AS-PCR mixture used in this study, Hotstart Taq DNA polymerase kits (BIONEER, South Korea), primer mixture, DNA template, and sterilized distilled water were added to make a total reaction volume of 50 µL . The primer mixture was added at the concentrations shown in Table 1. As shown in Table 2, the AS-PCR cycling used in this study was performed with a pre-denaturation step at 95 ${}^{\circ }$ C for 15 min, followed by 35 cycles of amplification; denaturation at 95 ${}^{\circ }$ C for 30 s, annealing for 30 s, and extension at 72 ${}^{\circ }$ C for 1 min. The final extension was performed at 72 ${}^{\circ }$ C for 5 min. The annealing temperatures were different for *HSPB1*, *ADH1C*, and *FASN* as follows: *HSPB1* 63 ${}^{\circ }$ C, *ADH1C* 61 ${}^{\circ }$ C, and *FASN* 61 ${}^{\circ }$ C. After AS-PCR, electrophoresis was performed at 100 V for 40 min in 0.5× tris-borate-EDTA buffer on 1.5 % agarose gels stained with ethidium bromide.

**Table 1:** Information, sequences, and concentrations of primers for SNPs used in this study.

Gene	SNP	Primer name	Primer sequence ( 5 – $3^{\prime }$ )	Concentration of primer
	(dbSNP no.)			in AS-PCR (uM)
		HSPB1-OF	GCACGGCTACATTTCCCGTTGCTTCAC	1.2
HSPB1	g.2352T > C	HSPB1-OR	TTACTTGTTTTCCGGCTGTTCGGACTTCCC	1.2
	(rs110832311)	HSPB1-IF	TAACTCTCGTCTACCCTCTTTGCCCGGCC ${}^{*}$	0.6
		HSPB1-IR	GTCCACACCGGGGGGCAGCATA ${}^{*}$	0.6
		ADH1C-OF	ACTGGTGTCTGATTTCTCTGTTGTGAAG	0.5
ADH1C	c.-64T > C	ADH1C-OR	AGAATTCCAGTTGAGCTATTCCAGATCC	0.5
	(rs110799147)	ADH1C-IF	AATCTGTGCAATCTATCTCTTGTATGTCCC ${}^{*}$	0.5
		ADH1C-IR	TTACAGACTTACAGGCTCTTCCCTGTTAAA ${}^{*}$	0.5
		FASN-OF	GGGAAATCCGGCAGCTCACAATCCACAA	0.8
FASN	g.17924G > A	FASN-OR	GTGTAGGCCATCACGAAGGTGTGCGAGC	0.8
	(rs41919985)	FASN-IF	CACCACCGTGTTCCACAGCCTGGACA ${}^{*}$	0.2
		FASN-IR	GGCCATAGGTGGGGATGCTGAGCTTTGC ${}^{*}$	0.2

${}^{*}$ Underlined means site of SNP.

**Table 2:** Different sizes of amplicons according to use of paired primers for different SNPs.

SNP	Genotype	Assay	Inter acting primers		Size of fragment (bp)
(dbSNP no.)			Forward	Reverse	
			HSPB1-OF		319
	CC	Variant type		HSBPB1-OR	
			HSPB1-IF		222
			HSPB1-OF		319
g.2352T > C				HSPB1-OR	
(rs110832311)	CT	Wild type and variant type	HSPB1-IF		222
			HSPB1-OF	HSPB1-IR	148
				HSPB1-OR	319
	TT	Wild type	HSPB1-OF		
				HSPB1-IR	148
			ADH1C-OF		762
	CC	Wild type		ADH1C-OR	
			ADH1C-IF		330
			ADH1C-OF		762
c.-64T > C				ADH1C-OR	
(rs110799147)	CT	Wild type and variant type	ADH1C-IF		330
			ADH1C-OF	ADH1C-IR	492
				ADH1C-OR	762
	TT	Variant type	ADH1C-OF		
				ADH1C-IR	492
			FASN-OF		830
	AA	Wild type		FASN-OR	
			FASN-IF		377
			FASN-OF		830
g.17924G > A				FASN-OR	
(rs41919985)	AG	Wild type and variant type	FASN-IF		377
			FASN-OF	FASN-IR	507
				FASN-OR	830
	GG	Variant type	FASN-OF		
				FASN-IR	507

### 2.3 DNA sequencing for the validation of genotyping

DNA sequencing was carried out to verify the AS-PCR results. The PCR product used for sequencing was amplified using a pair of outer primers for AS-PCR, and Labopass™ IP pro-Taq DNA polymerase (Cosmogenetech, South Korea). Sequencing was performed using an AllInOneCyclerTM 384 well PCR system (BIONEER, South Korea) using BigDye Terminator v3.1 sequencing kits (Applied Biosystems, USA), and then sequenced using an ABI3730XL (Applied Biosystems, USA).

## 3 Results and discussion

### 3.1 Association of identified SNPs with beef quality and quantity in Korean cattle

In this study, we used three SNPs identified by a previous study into the production of beef of high quality and quantity in Korean cattle. The SNPs were related to the production of high-quantity beef in Hanwoo. The g.2352T > C SNP in the *HSPB1* gene produced a significant difference in meat quantity, and the performance of animals with the CC homozygous genotype was higher than that of other genotypes (Suh et al., 2020). In a study into the production of high-quality beef, the CT heterozygote genotype of c.-64T > C SNP in the *ADH1C* gene was found to have an increased marbling score compared with the other genotypes (Peng et al., 2017). Oh et al. (2012) suggested that the marbling score of animals with the GG homozygous genotype of the g.17924G > A SNP in the *FASN* gene was significantly increased over those of other genotypes.

In order to analyze the genotype of these SNPs, methods such as PCR-RFLP and direct sequencing are widely used. However, these methods have several disadvantages. The experimental process is time-consuming and expensive, and the enzyme treatment generates analytical errors. We proposed an allele-specific polymerase chain reaction (AS-PCR), for analyzing genotypes without the need for enzyme treatment, to overcome these disadvantages.

### 3.2 AS-PCR for *HSPB1* g.2352T > C, *ADH1C* c.-64T > C and *FASN* g.17924G > A

In this study, we performed AS-PCR genotyping analysis using three SNPs *HSPB1* g.2352T > C, *ADH1C* c.-64T > C, and *FASN* g.17924G > A known to be related to beef quantity and quality. AS-PCR is a PCR-based method that uses specifically designed primers to permit amplification by DNA polymerase only if the nucleotide at the 3 ${}^{\prime }$ end of the primer perfectly binds to one complementary base in the variant or wild-type sequences. The expected fragment sizes following amplification using a primer pair designed for specific SNPs in the previous study are shown in Fig. 2. As shown in Fig. 2, two primer pairs were used in this study: the outer primer and the inner primer. In order to design the outer and inner primer for each SNP, DNA fragments which contained flanking DNA sequences of 1000 bp towards the 5 and 3 ${}^{\prime }$ directions from the SNP position were used. The sequence was designed to mismatch at the third base at the 3 ${}^{\prime }$ end of the inner primer, to increase the specificity for the alleles of the three SNPs (Liu et al., 2012). As shown in Table 1, the third base at the 3 ${}^{\prime }$ end of the inner primer was mismatched from T to G (*HSPB1*), A to C (*ADH1C*), and C to A (*FASN*).

In the first-round PCR, the amplified fragment using the outer primer of these SNPs was used as a positive control, and subsequently the fragment was distinguished by size, depending on the combination of outer and inner primer in the second-round PCR. The concentration of primer affected the amplification when using primer combinations in the AS-PCR method. The primer concentrations of the three SNPs used in this study are shown in Table 1.

The different sizes of fragments based on the combination of outer and inner primers are shown in Table 2 and Fig. 2. For the g.2352T > C SNP of *HSPB1* gene, the fragment size of the positive control was 319 bp, amplified by HSPB1-OF/HSPB1-OR. Based on the positive control, the HSPB1-IF/HSPB1-OR and HSPB1-OF/HSPB1-IR combinations amplified the C and T alleles to 222 and 148 bp, respectively. Therefore, as shown in Fig. 2, the g.2352T > C SNP of the *HSPB1* gene was found to be 148 and 319 bp in the TT homozygous genotype and 222 and 319 bp in the CC homozygous genotype. However, in the CT heterozygous genotype, all of the alleles for these SNPs (148, 222, and 319 bp) were amplified.

For the c.-64T > C SNP of the *ADH1C* gene, the positive control amplicon was 762 bp, when amplified by the ADH1C-OF/ADH1C-OR combination. Based on the positive control amplicon, the ADH1C-IF/ADH1C-OR and ADH1C-OF/ADH1C-IR combinations amplified the C and T allele to 330 and 492 bp, respectively. The c.-64T > C SNP of the *ADH1C* gene was identified as 330 and 492 bp alleles, amplified to 762 and 492 bp in the TT homozygous genotype, 762 and 330 bp in the CC homozygous genotype, and 330, 492, and 762 bp in the CT heterozygous genotype (Fig. 2b). Finally, in the g.17924G > A SNP of the *FASN* gene, FASN-IF/FASN-OR, and FASN-OF/FASN-IR amplified the A and G alleles to 377 and 507 bp based on the 830 bp amplicon. As shown in Fig. 2c, the A (377 bp) and G (507 bp) alleles were amplified to 507 and 830 bp in the GG homozygous genotype, 377 and 830 bp in the AA homozygous genotype, and 377, 507, and 830 bp in the AG heterozygous genotype.

Direct sequencing was performed to validate the AS-PCR results. The genotype predicted by the AS-PCR and the genotype confirmed through direct sequencing were consistent (Fig. 2d and e).

## 4 Conclusions

Currently, PCR-RFLP and the sequencing method, which are the most widely used for genotyping, use expensive chemical reagents such as restriction enzymes and fluorescent nucleic acid stains, also require professional labor and equipment, and are time-consuming due to complicated experimental process. Therefore, there is a need for a simple and accurate genotyping method that can be implemented in an environment without professional labor and sophisticated equipment. The AS-PCR method developed in this study requires only basic equipment: a thermal cycler and an electrophoresis system. Also it is cost-effective, because it uses only DNA polymerase and does not use restriction enzymes or fluorescent nucleic acid stains, and high sensitivity and specificity is provided by the inclusion of positive and negative controls according to principle that primers bind only to complementary sequences. This research team analyzed 3 SNPs related to meat quantity and quality in Korean cattle using AS-PCR method, and all 10 samples were amplified to match the expected product size according to the designed primers, achieving successful experimental results. Therefore, the use of the AS-PCR method will enable researchers to carry out genotyping analysis for SNPs (*HSPB1* g.2352T > C, *ADH1C* c.-64T > C, *FASN* g.17924G < A) without the use of sophisticated instrumentation and at low cost.

## Data Availability

The original data of the paper are available from the corresponding author upon request.
